# *VyMYB24* Integrates Antioxidant Defense and Cold Acclimation Networks to Enhance Freezing Tolerance in Chinese Wild Grape *Vitis yeshanensis* ‘Yanshan’

**DOI:** 10.3390/plants15152348

**Published:** 2026-07-30

**Authors:** Ruxin Gai, Yi Wang, Feifei Han, Beibei Li, Xiucai Fan, Ruijin Zhou, Guirong Li

**Affiliations:** 1 School of Horticulture and Landscape Architecture, Henan Institute of Science and Technology, Xinxiang 453003, China; newgrapes@163.com (R.G.); wangyikarin@163.com (Y.W.); qiushougrape@163.com (F.H.); bbl@hist.edu.cn (B.L.); 2 Zhengzhou Fruit Research Institute of Henan Province, Chinese Academy of Agricultural Sciences, Zhengzhou 450009, China; fanxiucai@caas.cn

**Keywords:** Chinese wild grape ‘Yanshan’, *VyMYB24* gene, tobacco, cold tolerance, plant development

## Abstract

Cold stress severely impairs grapevine (*Vitis vinifera* L.) growth, development, and productivity, necessitating the identification of elite cold-resistance genes for breeding tolerant cultivars. Chinese wild grape germplasm, particularly the endemic *Vitis yeshanensis* ‘Yanshan’ ecotype, represents a valuable reservoir of stress-resistance alleles with exceptional cold hardiness. In this study, we isolated an R2R3-MYB transcription factor gene *VyMYB24* from ‘Yanshan’ grape and systematically characterized its function in low-temperature responses. *VyMYB24* expression was rapidly and strongly induced by cold stress, with transcript levels peaking at 12 h after treatment. Heterologous overexpression of *VyMYB24* in transgenic tobacco (*Nicotiana benthamiana*) induced pronounced architectural changes. Transgenic plants exhibited a dwarf and compact stature with enhanced lateral branching, thickened stems, and robust root systems. In addition, anatomical analysis revealed markedly increased xylem and phloem thickness. Under cold stress, transgenic lines outperformed wild-type plants, with reduced wilting, lower water loss, and improved survival and recovery rates. Physiologically, *VyMYB24* activated the antioxidant defense system, elevated the activities of key reactive oxygen species (ROS)-scavenging enzymes, suppressed H_2_O_2_ and superoxide accumulation, and reduced malondialdehyde content and electrolyte leakage, thereby preserving cellular homeostasis. This study provides functional evidence for the positive regulatory role of *VyMYB24* in cold tolerance via heterologous expression, underscores the genetic value of ‘Yanshan’ grape germplasm, and lays a preliminary foundation for improving crop stress resistance through the utilization of native wild germplasm genes.

## 1. Introduction

Grapevine (*Vitis* spp.) is one of the most economically important fruit crops worldwide, cultivated extensively for fresh consumption, winemaking, and juice processing. Among cultivated grape species, *Vitis vinifera* L. dominates global viticulture due to its superior berry quality. However, its production is severely constrained by high susceptibility to abiotic stresses, with low temperature being a major limiting factor for geographic distribution and yield stability [1,2]. Cold stress, including chilling (0–15 °C) and freezing (<0 °C) temperatures, causes extensive cellular damage in plants, such as disruption of membrane integrity, inhibition of photosynthetic efficiency, and excessive accumulation of reactive oxygen species (ROS), ultimately impairing plant growth, development, and productivity [3]. Grapevine responses to abiotic stresses are highly dependent on cultivar-specific traits and environmental conditions. Nutrient status can further modulate stress physiological performance [4]; In addition, hormonal signaling networks, including jasmonic acid and abscisic acid pathways, have emerged as key mediators of secondary metabolite responses to stress [5,6]. These findings highlight the complexity of grapevine abiotic stress responses, which involve intricate interactions between genotype, environmental factors, and hormonal signaling pathways [3,4].

To cope with cold stress, plants have evolved sophisticated molecular mechanisms to perceive and transduce low-temperature signals, which involve the activation of complex transcriptional networks in which transcription factors (TFs) play central roles in orchestrating the expression of downstream stress-responsive genes [7]. The MYB TF family is one of the largest and most functionally diverse TF groups in plants, regulating a wide range of biological processes from development and secondary metabolism to stress adaptation [7]. In grapevine, 279 MYB genes have been identified, among which R2R3-MYB subfamily members are particularly well known for their involvement in secondary metabolism, cell differentiation, and stress signaling [8]. Accumulating evidence demonstrates the critical roles of R2R3-MYB TFs in modulating plant adaptation to environmental challenges. For example, in *Arabidopsis thaliana*, *AtMYB15* interacts with ICE1 to regulate the expression of cold-responsive genes [9]. In rice (*Oryza sativa*), *OsMYB4* and *OsMYB30* enhance chilling and freezing tolerance by modulating osmoprotectant metabolism and antioxidant defense [10,11]. In grape, accumulating studies have shown that bioactive substances can enhance plant stress tolerance by regulating antioxidant systems and secondary metabolic pathways such as phenylpropane metabolism [12,13]. These studies collectively illustrate the conserved yet diverse regulatory functions of R2R3-MYB TFs in plant cold acclimation [14].

China is a primary center of origin for grapevines and harbors an exceptionally rich and genetically diverse pool of wild grape germplasm, representing an invaluable source of stress-resistance alleles [15]. Among these native resources, the Chinese wild grape *V. yeshanensis* ‘Yanshan’ exhibits outstanding cold hardiness: its aerial canes can tolerate extreme temperatures as low as −35 °C, and its root systems remain viable at −14 °C to −16 °C. Notably, compared with other cold-tolerant wild species such as *Vitis amurensis*, ‘Yanshan’ grape possesses more desirable horticultural traits, making it an ideal genetic donor for targeted improvement of cold tolerance in cultivated grapevines [16,17].

Our previous work first isolated the R2R3-MYB transcription factor gene *VyMYB24* from *V. yeshanensis* ‘Yanshan’, and systematically confirmed its function in drought stress response and plant development regulation: *VyMYB24* is a nuclear-localized protein that can be significantly induced by drought; heterologous overexpression in tobacco causes a dwarf phenotype by negatively regulating gibberellin (GA) biosynthesis and enhances drought tolerance by promoting root development, activating the antioxidant enzyme system, and accumulating proline and up-regulating stress-responsive genes [18].

Given the widespread cold sensitivity of elite *V. vinifera* cultivars, mining and characterizing potent cold-tolerance genes from resilient wild germplasm is critical for sustainable viticulture [2,15]. Building on these published findings, the present study aimed to elucidate the functional role of *VyMYB24* in low-temperature stress responses. We systematically investigated the expression pattern of *VyMYB24* under cold stress, characterized the phenotypic and physiological effects of its heterologous overexpression in tobacco under low-temperature conditions, and dissected the mechanisms by which *VyMYB24* enhances cold tolerance. Our results demonstrate that *VyMYB24* functions as a positive regulator of cold tolerance through a dual mechanism: promoting anatomical and morphological adaptations that support stress resilience and activating antioxidant defenses to mitigate oxidative damage. This study advances our understanding of MYB-mediated cold signaling in perennial fruit crops and provides a valuable genetic resource for molecular breeding of cold-tolerant grape cultivars.

## 2. Results

### 2.1. Generation of VyMYB24-Overexpressing Transgenic Tobacco Plants

**Figure 1:**
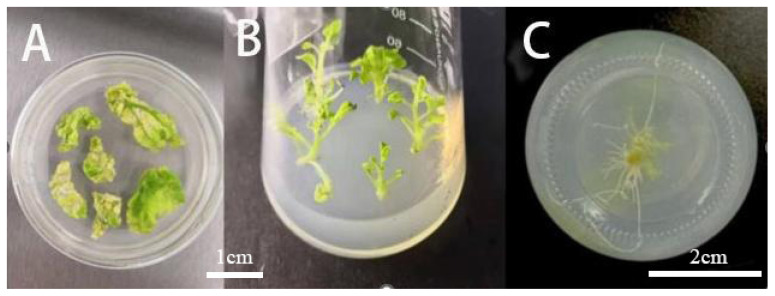
Stepwise in vitro regeneration of *VyMYB24*-overexpressing transgenic *Nicotiana benthamiana* via *Agrobacterium tumefaciens*-mediated leaf disc transformation. (**A**) Leaf explants co-cultivated with Agrobacterium and cultured on kanamycin-containing selective medium; pale yellow compact resistant calli were induced at leaf wound edges under antibiotic screening pressure; (**B**) Massive adventitious shoot differentiation from kanamycin-resistant callus after continuous subculture on regeneration medium; (**C**) Complete regenerated plantlets with intact root systems obtained after 14 d of rooting induction on rooting medium. Scale bars: (**A**,**B**) 1 cm; (**C**) 2 cm. All selective media were supplemented with 50 mg·L^−1^ kanamycin for transformant screening.

After 3 d of dark co-cultivation with Agrobacteria, leaf discs were transferred to selective regeneration medium containing kanamycin. Under continuous antibiotic selective pressure, untransformed leaf tissues gradually bleached and senesced, whereas the wound margins of positive explants underwent active dedifferentiation and produced compact, pale yellow kanamycin-resistant callus masses (Figure 1A). Subculture of resistant calli onto fresh differentiation medium facilitated continuous cell division and organogenesis, with abundant adventitious shoot primordia emerging and differentiating from the surface of callus clumps (Figure 1B). Robust, well-developed adventitious shoots with intact leaf primordia were excised and transferred to hormone-supplemented rooting medium for root induction. After approximately 14 d of culture, complete root systems were successfully differentiated from shoot bases, generating whole regenerated plantlets that were ready for acclimatization and greenhouse transplantation (Figure 1C). The tissue culture and regeneration pipeline used in this study was identical to the stable system established in our previous study on *VyMYB24* [18], with highly repeatable efficiency in callus induction, adventitious shoot differentiation, and rooting, enabling the bulk acquisition of kanamycin-resistant regenerated seedlings with a consistent genetic background.

Following regeneration, molecular identification was performed on all resistant lines using the same verified protocol as our previous work [18]. Genomic PCR amplification confirmed the integration of the 35S:*VyMYB24* expression cassette into the tobacco genome, with no target band detected in wild-type (WT) plants. qRT-PCR further verified that *VyMYB24* was stably expressed in transgenic lines, among which OE1 and OE3 showed relatively high expression levels. Two independent T_2_ homozygous lines (OE1, OE3) with stable high expression were screened via a kanamycin resistance segregation ratio assay combined with qRT-PCR verification, and selected for subsequent functional assays. The molecular characteristics and expression levels of the two transgenic lines were consistent with those used in our previous drought stress study [18], ensuring the uniformity of experimental materials across different stress assays. This robust explant regeneration system provided sufficient transgenic materials for phenotypic and physiological functional characterization of *VyMYB24*.

### 2.2. VyMYB24 Is Rapidly and Strongly Induced by Low-Temperature Stress

**Figure 2:**
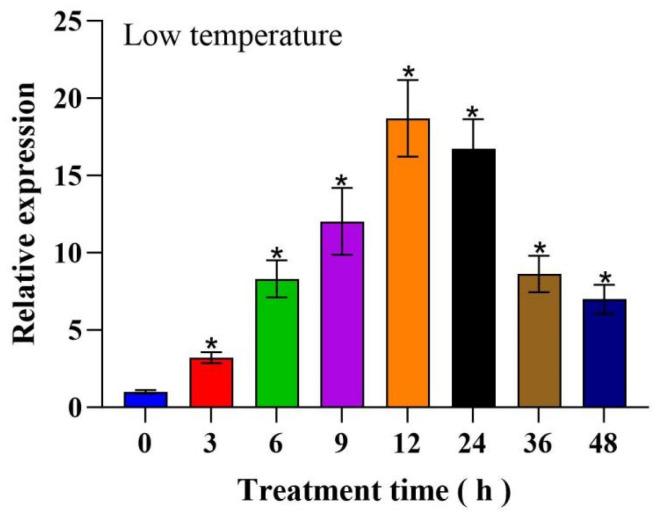
Expression profiles of *VyMYB24* in *V. yeshanensis* ‘Yanshan’ in response to low-temperature stress. Relative transcript levels of *VyMYB24* were detected by qRT-PCR in leaves exposed to 0 °C for 0, 3, 6, 9, 12, 24, 36 and 48 h. *Vitis* Actin was used as the endogenous reference gene for normalization. Data were presented as mean ± standard deviation (SD). * indicated statistically significant differences compared with the 0 h control group at *p* < 0.05.

qRT-PCR analysis showed that *VyMYB24* expression in *V. yeshanensis* ‘Yanshan’ leaves exhibited a rapid and sustained induction pattern under 0 °C stress (Figure 2). Within the first 12 h of cold treatment, *VyMYB24* transcript levels increased continuously with stress duration, reaching a peak at 12 h with 20.63-fold upregulation relative to the untreated control (0 h). After 12 h, *VyMYB24* expression gradually declined but remained significantly higher than the control throughout the 48 h treatment period. Differences were assessed by one-way ANOVA followed by Tukey’s multiple comparison test, with *p* < 0.05 considered statistically significant. This expression profile indicates that *VyMYB24* is an early-responsive gene in the grape low-temperature response and may function as an upstream regulatory node in cold signaling.

### 2.3. VyMYB24 Overexpression Remodels Plant Architecture and Root Development

**Figure 3:**
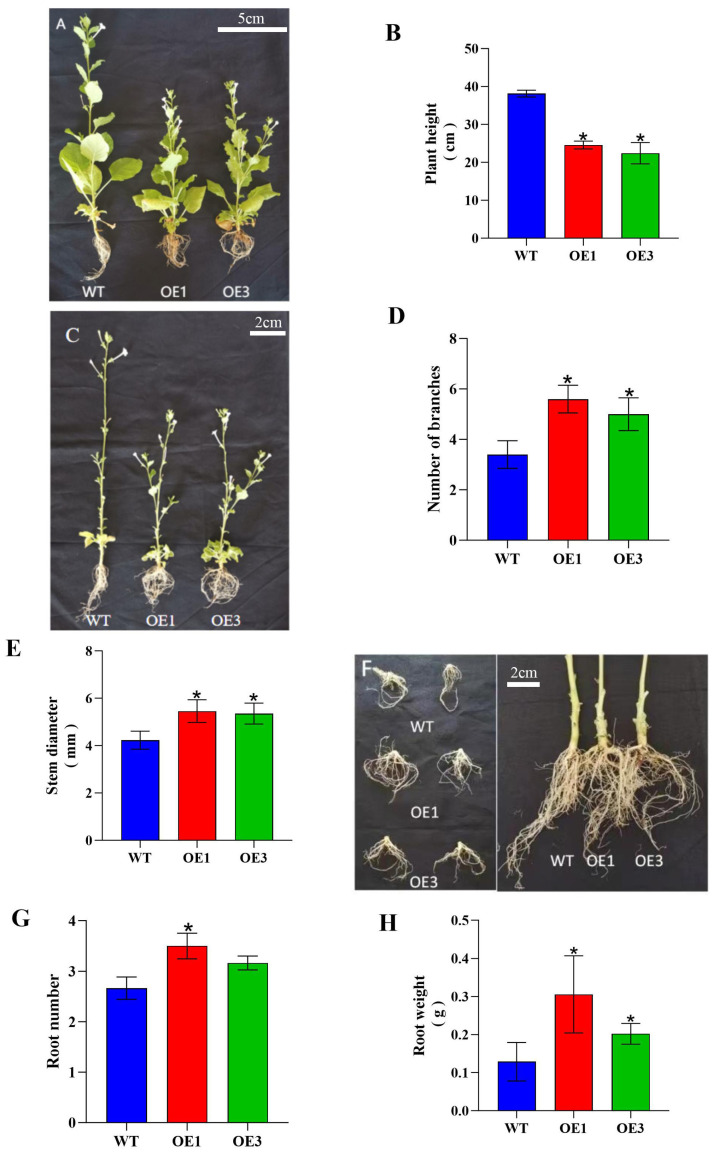
*VyMYB24* overexpression remodels plant architecture and root development in transgenic tobacco. (**A**) Overall growth phenotype of WT and *VyMYB24*-overexpressing (OE1, OE3) tobacco plants at 8 weeks after sowing; (**B**) Quantitative analysis of plant height; (**C**) Phenotypic comparison of lateral branches; (**D**) Statistical results of lateral branch number per plant; (**E**) Measurement of stem diameter; (**F**) Root morphology of WT and transgenic lines; (**G**) Quantification of root number per plant; (**H**) Statistical analysis of root fresh weight. Scale bars: (**A**) 5 cm; (**C**,**F**) 2 cm. Data were presented as mean ± SD. * indicated statistically significant differences compared with the wild-type (WT) group at *p* < 0.05.

Under normal growth conditions, *VyMYB24*-overexpressing tobacco lines (OE1, OE3) exhibited significant remodeling of plant architecture, stem morphology, and root development compared with WT plants (Figure 3A). At 8 weeks after sowing, the plant heights of OE1 and OE3 were only 64.45% and 58.73% of WT, respectively, showing a prominent dwarf phenotype (Figure 3B). The number of lateral branches increased significantly, reaching 1.65-fold and 1.47-fold of WT, resulting in a compact and bushy plant architecture (Figure 3C,D).

Stem diameter measurements showed that OE1 and OE3 stems were 28.99% and 26.55% thicker than WT, respectively, indicating enhanced mechanical support (Figure 3E). Underground root development showed even more pronounced differences: the root numbers of OE1 and OE3 lines were 137.5% and 127.1% of WT, respectively (Figure 3F,G), and the root fresh weights of OE1 and OE3 reached 2.38-fold and 1.57-fold that of WT, respectively, representing significantly increased root biomass (Figure 3H).

These results indicate that *VyMYB24* coordinately regulates aboveground architecture and underground root development in tobacco, inducing a stress-adaptive phenotype with dwarf compact stature, thick stems, and robust root systems. This structural remodeling provides morphological support for water and nutrient uptake as well as mechanical stability under low-temperature stress.

### 2.4. VyMYB24 Overexpression Promotes Anatomical Remodeling of Stem Vascular Tissues

**Figure 4:**
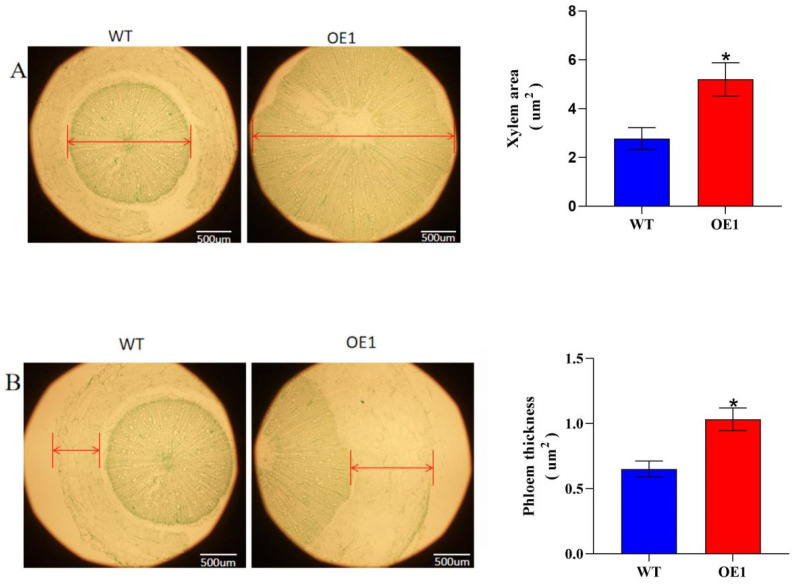
Anatomical analysis of stem vascular tissues in *VyMYB24*-overexpressing tobacco plants. (**A**) Xylem morphology in stem cross-sections (left panel) and quantitative measurement of xylem thickness (unit: μm, right panel); (**B**) Phloem morphology in stem cross-sections (left panel) and quantitative measurement of phloem thickness. Scale bars = 500 μm. Data were presented as mean ± SD. * indicated statistically significant differences compared with the wild-type (WT) group at *p* < 0.05.

Examination of paraffin cross-sections of stems after low-temperature stress showed that *VyMYB24* overexpression significantly regulated stem vascular tissue development in tobacco, with synchronous thickening of xylem and phloem (Figure 4). Microscopic observation revealed wider vascular tissue regions in transgenic lines compared with WT, with more differentiated vascular tissues and tighter cell arrangement.

Quantitative analysis using ImageJ2026 1.8.0 showed that xylem thickness in OE1 and OE3 lines was 1.87-fold and 1.79-fold that of WT, respectively (Figure 4A), and phloem thickness was 1.58-fold and 1.52-fold that of WT, respectively (Figure 4B), with both differences reaching statistical significance.

The enhanced vascular system improves both mechanical support and water/nutrient transport efficiency, which serves as an inherent structural basis for the improved low-temperature tolerance of transgenic lines.

### 2.5. Overexpression of VyMYB24 Enhances Chilling Tolerance and Freezing Survival in Tobacco Seedlings

**Figure 5:**
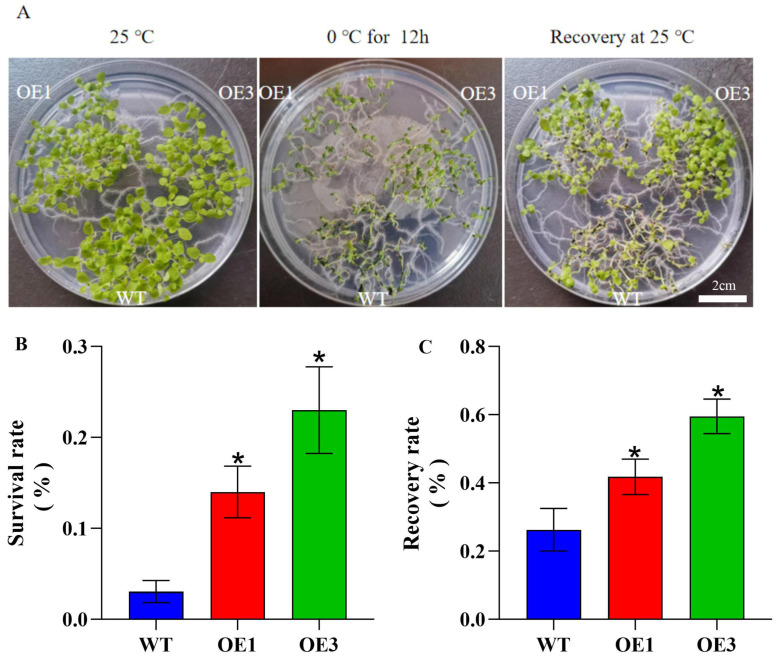
Overexpression of *VyMYB24* enhances chilling and freezing tolerance in transgenic tobacco seedlings. (**A**) Representative phenotypes of wild-type (WT) and *VyMYB24*-overexpressing (OE1, OE3) tobacco seedlings under normal growth conditions (25 °C), after 0 °C chilling stress for 3 d, and after 3 d of recovery at 25 °C. Scale bar = 2 cm; (**B**) Survival rate of tobacco seedlings after extreme freezing treatment at −20 °C for 2 h. Survival was defined as seedlings retaining green, viable shoot apical meristems and capable of regrowth after recovery; (**C**) Recovery rate of tobacco seedlings after 3 d of regrowth under normal conditions following 0 °C chilling stress. Data were presented as mean ± SD. * indicated statistically significant differences compared with the wild-type (WT) group at *p* < 0.05.

To evaluate the regulatory role of *VyMYB24* in overall plant cold tolerance, 4-week-old tobacco seedlings of WT and transgenic lines with uniform growth were selected for whole-plant low-temperature stress treatment. Under normal growth conditions (25 °C), no obvious phenotypic differences were observed between WT and transgenic seedlings. After 3 days of continuous chilling treatment at 0 °C, WT seedlings exhibited severe leaf wilting, chlorosis and drooping, with necrotic lesions on leaf margins. In contrast, OE1 and OE3 transgenic seedlings only showed slight leaf wilting and maintained a relatively intact overall morphology (Figure 5A). Statistical analysis showed that the wilting rates of OE1 and OE3 lines were 26% and 23%, respectively, which were significantly lower than that of WT (*p* < 0.05).

To further verify tolerance to extreme freezing stress, seedlings were exposed to −20 °C for 2 h and then returned to normal conditions for recovery. The results showed that the survival rate of WT seedlings was only 8%, with most plants turning completely chlorotic and necrotic after freezing. By contrast, the survival rates of the OE1 and OE3 transgenic lines reached 41% and 47%, respectively, representing a >5-fold significant increase relative to the 8% survival rate of wild-type plants (Figure 5B). After 3 days of recovery at 25 °C following 0 °C chilling stress, transgenic lines also showed significantly better recovery capacity, with recovery rates 3.2- and 4.3-fold higher than that of WT for OE1 and OE3, respectively (Figure 5C). Taken together, these results demonstrate that overexpression of *VyMYB24* simultaneously improves chilling tolerance, freezing survival and post-stress recovery potential in tobacco seedlings.

### 2.6. Overexpression of VyMYB24 Preserves Leaf Plasma Membrane Integrity Under Cold Stress

**Figure 6:**
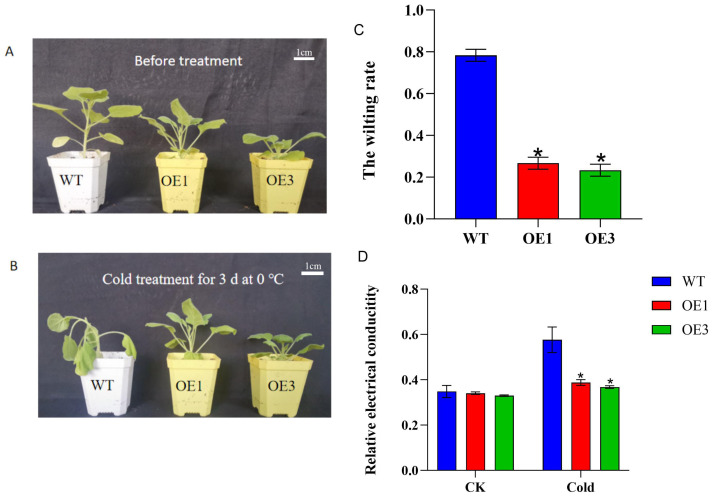
Overexpression of *VyMYB24* alleviates cold-induced plasma membrane damage in tobacco leaves. (**A, B**) Representative phenotypes of wild-type (WT) and *VyMYB24*-overexpressing transgenic lines (OE1, OE3) before cold treatment (**A**) and after 3 days of 0 °C chilling stress (**B**). Scale bar = 1 cm. (**C**) Wilting rate of WT and transgenic lines after 3 days of 0 °C chilling stress. (**D**) Relative electrical conductivity of leaves from WT and transgenic lines under normal growth conditions (CK) and after 3 days of 0 °C chilling stress. Data were presented as mean ± SD. * indicated statistically significant differences compared with CK group at *p* < 0.05.

Low-temperature stress primarily damages plant cells by disrupting membrane structure. To clarify the alleviating effect of *VyMYB24* on cold-induced cellular injury in leaves, fully expanded functional leaves from 8-week-old mature plants with uniform growth status were collected for in vitro cold treatment. Under normal temperature conditions, leaves from both WT and transgenic lines were fully expanded with normal color, showing no significant difference (Figure 6A). After 3 days of cold treatment at 0 °C, WT leaves showed obvious water loss, shrinkage, darkening and tissue texture damage, whereas transgenic leaves remained relatively turgid with milder injury (Figure 6B).

Relative electrolyte leakage is a core physiological indicator reflecting plasma membrane integrity. Under normal conditions, relative electrolyte leakage remained at a low level in all lines with no significant difference. After 3 days of 0 °C cold treatment, relative electrolyte leakage increased significantly in all lines. Specifically, relative electrolyte leakage in WT leaves rose to 57%, indicating severe membrane damage and massive intracellular ion leakage. In contrast, the values in OE1 and OE3 transgenic lines were only 38% and 36%, respectively, which were significantly lower than that of WT (*p* < 0.05) (Figure 6C). Consistent with the leaf phenotypes, these results indicate that *VyMYB24* overexpression effectively mitigates cold-induced plasma membrane damage and maintains cellular homeostasis.

### 2.7. Effects of VyMYB24 Overexpression on Excessive ROS Accumulation in Tobacco Under Low-Temperature Stress

**Figure 7:**
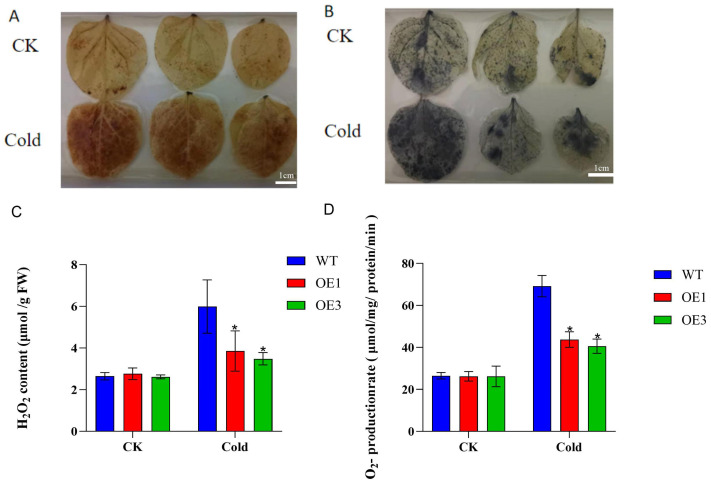
*VyMYB24* overexpression suppresses excessive ROS accumulation in tobacco under low-temperature stress. (**A**) Histochemical detection of hydrogen peroxide (H_2_O_2_) via DAB staining; (**B**) Histochemical detection of superoxide anion (O_2_·^−^) via NBT staining; (**C**) Quantitative measurement of H_2_O_2_ content in leaf tissues; (**D**) Determination of O_2_·^−^ production rate in leaves. All assays were conducted after 3 days of 0 °C cold treatment. Scale bars in (**A**,**B**) = 1 cm. Data were presented as mean ± SD. * indicated statistically significant differences compared with CK group at *p* < 0.05.

Under normal growth conditions, 3,3′-diaminobenzidine (DAB) and nitroblue tetrazolium (NBT) staining of leaves from both WT and transgenic plants were faint, with no significant difference in basal ROS (including H_2_O_2_ and O_2_·^−^) accumulation. After 3 days of low-temperature stress, WT leaves showed dark brown DAB staining (indicating massive H_2_O_2_ accumulation) and dark blue NBT staining (indicating massive O_2_·^−^ accumulation), whereas transgenic leaves showed extremely faint staining with significantly reduced ROS levels (Figure 7A,B).

Quantitative ROS measurements were consistent with staining results: after low-temperature stress, the H_2_O_2_ content in WT was 64.4% and 64.4% higher than that in OE1 and OE3, respectively, and the O_2_·^−^ production rate was 63.2% higher on average compared with the transgenic lines (Figure 7C,D). These results indicate that *VyMYB24* overexpression effectively inhibits the low-temperature-induced ROS burst, maintains cellular redox homeostasis, and reduces oxidative damage caused by excessive ROS accumulation.

### 2.8. VyMYB24 Overexpression Enhances Antioxidant Enzyme Activities and Alleviates Membrane Lipid Peroxidation

**Figure 8:**
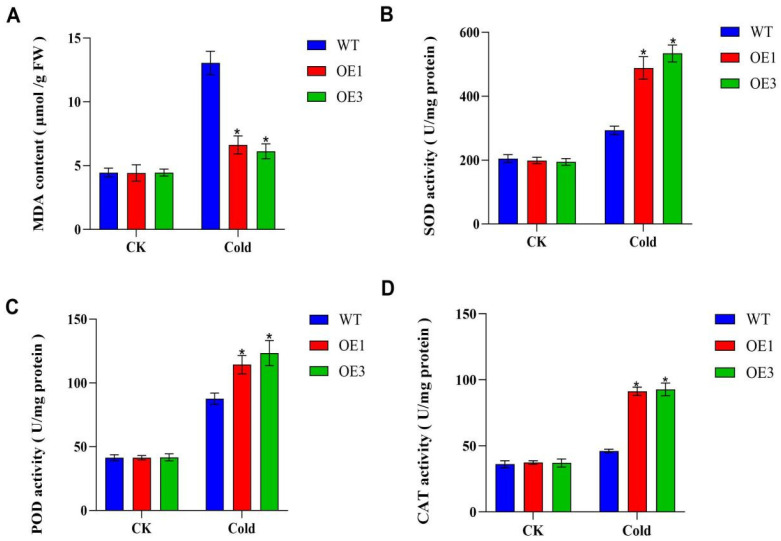
Overexpression of *VyMYB24* enhances antioxidant enzyme activities and reduces membrane lipid peroxidation under cold stress. (**A**) MDA content, a hallmark product of membrane lipid peroxidation reflecting oxidative damage degree; (**B**) SOD enzyme activity; (**C**) POD enzyme activity; (**D**) CAT enzyme activity. Data were presented as mean ± SD. * indicated statistically significant differences compared with CK group at *p* < 0.05.

Malondialdehyde (MDA) is a hallmark product of membrane lipid peroxidation, and its content directly reflects the degree of oxidative damage to cell membranes. After low-temperature stress, MDA contents in OE1 and OE3 were only 49.24% and 53.13% of WT, respectively, indicating greatly reduced membrane lipid peroxidation (Figure 8A).

Antioxidant enzyme activity assays showed that under normal growth conditions, there was no significant difference in superoxide dismutase (SOD), peroxidase (POD), or catalase (CAT) activities between transgenic and WT plants. After low-temperature stress, however, antioxidant enzyme activities in transgenic lines increased extremely significantly: SOD activities in OE1 and OE3 were 1.82-fold and 1.66-fold of those in WT, respectively, with a significant difference (*p* < 0.05) (Figure 8B), POD activities were 1.41-fold and 1.30-fold of WT (Figure 8C), and CAT activities reached 2.01-fold and 1.98-fold of WT (Figure 8D).

These results indicate that *VyMYB24* enhances the antioxidant enzyme system under low-temperature stress, efficiently scavenging excess ROS, reducing membrane lipid peroxidation damage, maintaining cell membrane integrity and normal physiological function, thereby improving plant low-temperature tolerance.

## 3. Discussion

In this study, we isolated *VyMYB24*, an R2R3-MYB transcription factor, from the highly cold-hardy Chinese wild grape *V. yeshanensis* ‘Yanshan’, and systematically characterized its function and regulatory mechanism in response to low-temperature stress. Our findings confirm that *VyMYB24* acts as an early-responsive node in cold signaling and synergistically enhances plant cold tolerance through a dual regulatory pathway mediating plant morphological remodeling and the activation of the antioxidant defense system. This study provides new insights into the role of MYB genes in cold stress regulation in *V. yeshanensis* and offers elite wild germplasm gene resources for molecular breeding of cold-tolerant grapevines.

Expression pattern analysis revealed that *VyMYB24* was rapidly and persistently induced by low temperature, with its transcript level peaking at 12 h under 0 °C treatment. This expression pattern is highly consistent with previously reported cold-tolerant MYB genes across multiple plant species. In *A. thaliana*, *AtMYB15*, a key component of the cold signaling pathway, is rapidly induced by low temperature and mediates plant cold acclimation by interacting with ICE1 to regulate the expression of downstream CBF genes [9]. Similarly, *MdMYB23* in apple is significantly upregulated under low temperature and positively regulates plant freezing tolerance by activating the CBF-dependent pathway [12]. *OsMYB3R-2* in rice also exhibits early induction by low temperature and plays a crucial role in abiotic stress responses [19]. In grape, exogenous melatonin has been proven to alleviate low-temperature damage during postharvest storage by activating antioxidant metabolism [20]. The rapid induction kinetics suggest that *VyMYB24* may function as an upstream regulatory factor in the grape cold response cascade. Whether *VyMYB24* participates in the canonical inducer of CBF expression (ICE)-C-repeat binding factor (CBF) cold signaling pathway requires further verification via molecular interaction assays.

Tobacco plants heterologously overexpressing *VyMYB24* exhibited morphological characteristics, including dwarfism, increased lateral branching, thickened stems, and elevated root biomass. Meanwhile, xylem and phloem thickness in stem cross-sections was significantly increased in both transgenic lines, indicating a reinforced vascular structure. This morphological remodeling is closely associated with improved plant stress tolerance: compact plant architecture enhances overall freezing resilience by reducing transpirational water loss (which worsens cellular dehydration) and minimizing surface area susceptible to ice crystal-induced mechanical damage, while a well-developed root system and reinforced vascular system enhance the absorption and transport efficiency of water and nutrients, providing a structural basis for the maintenance of physiological homeostasis under stress.

Consistent with the morphological phenotypes observed in our previous drought study [18], it is speculated that *VyMYB24* modulates plant architecture by repressing GA biosynthetic genes and upregulating GA degradative genes to reduce endogenous GA levels, and the dwarf phenotype can be restored by exogenous GA application. This regulatory mechanism is conserved across plant species, as R2R3-MYB transcription factors are widely documented to participate in gibberellin and brassinosteroid signaling pathways and control plant morphogenesis via regulating cell elongation and division [14]. This study further demonstrates that this GA-mediated morphological remodeling also serves as a critical structural adaptation under low-temperature stress, indicating that the developmental regulatory module of *VyMYB24* acts as a shared adaptive mechanism in response to multiple abiotic stresses.

Low-temperature stress triggers excessive accumulation of reactive oxygen species (ROS) in plant cells, which drives membrane lipid peroxidation, compromises plasma membrane integrity, and ultimately causes oxidative damage and cell death [21]. In this study, overexpression of *VyMYB24* markedly suppressed the cold-induced burst of H_2_O_2_ and superoxide anions, reduced MDA content and relative electrolyte leakage, and simultaneously elevated the activities of key antioxidant enzymes, including SOD, POD and CAT.

This regulatory mode aligns with the conserved function of R2R3-MYB transcription factors across plant species. For instance, *OsMYB4* overexpression in rice enhances chilling tolerance by upregulating antioxidant gene expression and strengthening ROS scavenging capacity [10]; this antioxidant regulatory pattern is also widely confirmed in grape stress response studies [13]. Notably, our previous work has demonstrated that *VyMYB24* also activates the antioxidant defense system to mitigate oxidative damage under drought stress [18]. Combined with the cold-responsive phenotypes observed here, this finding indicates that the antioxidant regulatory module mediated by *VyMYB24* is not specific to a single stress, but acts as a shared adaptive mechanism underlying both drought and cold stress responses. Consistently, brassinosteroids, a class of plant growth regulators, have also been found to synergistically modulate grape growth and stress adaptation with transcription factor networks, which supports the pleiotropic regulatory function of *VyMYB24* [17]. Together with the findings of this study, these results collectively confirm the pleiotropic roles of *VyMYB24* in the positive regulation of multiple abiotic stress responses, which is a common functional feature of R2R3-MYB transcription factors [14].

This study reveals the molecular mechanism by which *VyMYB24* regulates grape cold tolerance through the dual module of “morphological remodeling–antioxidant defense.” Future studies can further verify its function in its native species via stable genetic transformation in grapevines, and deeply explore its downstream regulatory network, so as to provide theoretical support for molecular design breeding of cold-tolerant grapevines.

## 4. Materials and Methods

### 4.1. Plant Materials and Reagents

Two-year potted seedlings of cold-resistant *Vitis yeshanensis* ‘Yanshan’ were sourced from the Grape Germplasm Repository of Henan Institute of Science and Technology. Wild-type (WT) *Nicotiana benthamiana* seeds were preserved in our laboratory. All plant cultivation and low-temperature treatments were conducted in programmable artificial climate chambers (RXZ-1000, Ningbo Jiangnan Instrument Co., Ltd., Ningbo, China) with a 16 h light/8 h dark photoperiod, 300 μmol·m^−2^·s^−1^ light intensity, and 60–70% relative humidity. Control plants were grown under the same photoperiod and light conditions at a constant 25 °C.

*Escherichia coli* DH5α and *Agrobacterium tumefaciens* GV3101 strains, as well as the plant binary vector pCAMBIA2300, were maintained in our lab. Restriction endonucleases KpnI, SalI and T4 DNA ligase were purchased from Thermo Fisher Scientific (Waltham, MA, USA). Total RNA extraction kits were obtained from Omega Bio-tek (Norcross, GA, USA). PrimeScript™ RT Reagent Kit and SYBR Premix Ex Taq™ II were supplied by Takara (Dalian, China). Commercial detection kits for H_2_O_2_, O_2_·^−^, MDA, SOD, POD and CAT were purchased from Solarbio Science & Technology Co., Ltd. (Beijing, China). All other analytical-grade chemical reagents were commercially available in China.

### 4.2. Experimental Methods

Part of the experimental methods involved in this study, including vector construction, *Agrobacterium-mediated* tobacco genetic transformation, histochemical staining of ROS, and determination of antioxidant enzyme activity and membrane damage indicators, were consistent with our previously published research on *VyMYB24* under drought stress [18]. The specific operation details of the following experiments are described as follows.

#### 4.2.1. Experimental Design

(1) Cold-induced expression analysis of *VyMYB24*

Uniform 2-year-old potted ‘Yanshan’ grape seedlings were placed in a programmable growth chamber at 0 °C with a 16 h light/8 h dark photoperiod, 280 μmol·m^−2^·s^−1^ light intensity and 60% relative humidity for cold treatment. The 3rd fully expanded leaf from the shoot apex was harvested at 0, 3, 6, 9, 12, 24, 36 and 48 h after treatment, snap-frozen in liquid nitrogen and stored at −80 °C for subsequent RNA extraction. Three biological replicates were set for each time point. Seedlings were watered with 1/2 Hoagland nutrient solution once a week to maintain a consistent nutrient supply.

(2) Whole-plant cold tolerance assay of seedlings

Four-week-old WT and T_2_ transgenic tobacco (OE1, OE3) seedlings with uniform growth were transplanted into 7 cm (diameter) × 8 cm (height) plastic pots filled with a 3:1 (*v*/*v*) mixture of peat moss and vermiculite, and divided into control and treatment groups. The control group was cultured under normal conditions at 25 °C with a 16 h light/8 h dark photoperiod, 300 μmol·m^−2^·s^−1^ light intensity and 65% relative humidity. For chilling stress treatment, seedlings were placed in a 0 °C growth chamber under the same photoperiod, light intensity and humidity conditions for 3 consecutive days, followed by phenotypic observation and wilting rate calculation. Another batch of seedlings was exposed to −20 °C for 2 h for survival rate statistics. After cold stress, seedlings were transferred back to 25 °C for 3 days of recovery culture, and the recovery rate was calculated. Three biological replicates were set for each line, with 30 seedlings per replicate.

(3) Cold injury assay of mature leaves

Fully expanded functional leaves (3rd–4th from the top) with uniform growth were sampled from 8-week-old WT and transgenic tobacco plants grown under 25 °C, 16 h light/8 h dark photoperiod, 300 μmol·m^−2^·s^−1^ light intensity and 65% relative humidity, and watered weekly with 1/2 Hoagland nutrient solution. The detached leaves were placed in Petri dishes lined with moist filter paper. The control group was incubated at 25 °C in the dark, and the treatment group was incubated in the dark at 0 °C for 3 days. After treatment, leaf phenotypes were recorded and samples were collected for relative electrolyte leakage measurement. Three biological replicates were set for each line.

(4) Physiological and biochemical index determination

Tobacco leaves after 3 days of 0 °C treatment were collected for measurement of ROS content, antioxidant enzyme activities, MDA content and other physiological indicators, with leaves cultured at normal temperature as the control.

#### 4.2.2. Index Measurements

(1) Quantitative real-time PCR (qRT-PCR)

Total RNA was extracted using the E.Z.N.A.^®^ Plant RNA Kit (Omega Bio-tek, Norcross, GA, USA). RNA integrity was assessed by agarose gel electrophoresis, and RNA purity (OD260/OD280 = 1.8–2.0) was verified using a NanoDrop 2000 spectrophotometer (Thermo Fisher Scientific, Waltham, MA, USA). One microgram of qualified total RNA was reverse-transcribed into cDNA using the PrimeScript™ RT Reagent Kit (Takara Bio Inc., Otsu, Shiga, Japan). Quantitative real-time PCR (qRT-PCR) was performed on the LightCycler^®^ 480 real-time PCR system (Roche Diagnostics, Mannheim, Germany) with SYBR^®^ Premix Ex Taq™ II (Takara Bio Inc., Otsu, Shiga, Japan). Grape *Actin* and tobacco *Actin* were applied as internal reference genes, and relative transcript abundance was calculated by the 2^-ΔΔCT^ method [22]. Three biological replicates with three technical replicates were set for each sample.

(2) Vector construction and Agrobacterium-mediated transformation

Specific primers containing KpnI and SalI restriction sites were designed to amplify the full-length CDS of *VyMYB24*. Purified target fragments were double-digested and ligated into linearized pCAMBIA2300 to generate the 35S::*VyMYB24* overexpression vector. Recombinant plasmids were transformed into *A. tumefaciens* GV3101 by the freeze–thaw method, and positive bacterial clones were verified by PCR.

The classic leaf disc transformation approach was adopted to generate transgenic tobacco [23]. The selection medium was supplemented with 50 mg·L^−1^ kanamycin and 300 mg·L^−1^ cefotaxime to screen positive explants. After successive self-pollination and dual identification (genomic PCR + qRT-PCR), two stable homozygous T_2_ transgenic lines OE1 and OE3 were selected for subsequent functional assays.

(3) Morphological and cold-tolerance phenotypic statistics

Seedling wilting rate: percentage of plants with >50% leaf wilting area relative to the total number of plants.

Seedling survival rate: After 3 days of recovery following −20 °C freezing treatment, plants with green shoot apical meristems capable of regrowth were defined as surviving, and the survival rate was calculated.

Seedling recovery rate: After 3 days of recovery following 0 °C chilling stress, plants that regenerated new leaves and resumed normal growth were defined as recovered, and the recovery rate was calculated.

Morphological indices: for tobacco plants grown for 8 weeks under normal conditions, at least 10 plants per line were randomly selected to determine plant height, lateral branch number, stem diameter, root fresh weight and other morphological parameters.

(4) Histochemical staining and quantitative determination of ROS

In situ accumulation of H_2_O_2_ and O_2_·^−^ in tobacco leaves was visualized by DAB and NBT histochemical staining, respectively, following the published protocol [24]. Stained leaves were decolorized with ethanol and photographed. Meanwhile, leaf H_2_O_2_ content and O_2_·^−^ production rate were quantified using matching Solarbio detection kits in accordance with manufacturer instructions. All samples were collected after 3 d of 0 °C cold treatment.

(5) Cell membrane damage and antioxidant enzyme activity assays

Relative electrolyte leakage was determined using a DDS-308A conductivity meter to evaluate plasma membrane integrity [25]. Malondialdehyde (MDA) content, a marker of lipid peroxidation, was measured via the thiobarbituric acid color reaction [26].

SOD, POD and CAT activities were detected by NBT photoreduction, guaiacol oxidation and ultraviolet absorption methods, respectively [27,28], using commercial Solarbio kits. All physiological indicators were measured with three independent biological replicates.

(6) Paraffin section observation of stem vascular tissues

The 3rd internode of tobacco stems was sampled after cold stress, fixed in FAA fixative, dehydrated in graded ethanol, cleared with xylene and embedded in paraffin. Continuous 8 μm cross-sections were prepared and stained with safranin-fast green [29]. Vascular tissue structures were observed and photographed under an Olympus light microscope. The thickness of xylem and phloem was quantified using ImageJ2026 1.8.0 software [30], with at least 3 biological replicates per genotype and 10 random visual fields measured per replicate.

### 4.3. Data Processing and Statistical Analysis

All data are presented as mean ± standard deviation (SD). For raw data processing, quantitative real-time PCR fluorescence signals were acquired and initially analyzed with LightCycler^®^ 480 Software (version 1.5.0; Roche Diagnostics, Mannheim, Germany). The xylem and phloem thickness in stem paraffin cross-sections was measured and quantified using ImageJ software (version 1.8.0; National Institutes of Health, Bethesda, MD, USA). Statistical analyses were performed by one-way analysis of variance (ANOVA) using SPSS software (version 22.0; IBM Corp., Armonk, NY, USA). For the time-course expression analysis of *VyMYB24* under cold stress, Dunnett’s post-hoc test was applied to compare the relative transcript level at each time point with that of the 0 h control. For phenotypic, physiological and biochemical assays, Dunnett’s post-hoc test was used to compare transgenic lines (OE1, OE3) with the wild-type (WT) group under identical growth or stress conditions. Differences were considered statistically significant at *p* < 0.05, * are used to denote significant differences in all figures. All statistical charts in this study were generated using OriginPro software (version 2021; OriginLab Corp., Northampton, MA, USA).

## 5. Conclusions

This study systematically characterized the function and regulatory mechanism of *VyMYB24*, an R2R3-MYB transcription factor isolated from the cold-tolerant Chinese wild grape *V. yeshanensis* ‘Yanshan’. As an early-responsive gene to low-temperature stress, *VyMYB24* expression is rapidly and strongly induced, peaking at 12 h post-treatment, underscoring its pivotal role in the initial cold stress response. Heterologous expression of *VyMYB24* in tobacco not only reshapes plant architecture—manifesting as dwarfism, increased lateral branching, thickened stems, and a robust root system—but also promotes vascular tissue development, with significant thickening of xylem and phloem, thereby enhancing structural support and resource transport capacity. Under cold stress, *VyMYB24*-overexpressing lines exhibit superior cold tolerance compared with wild-type plants, characterized by lower wilting rates, reduced electrolyte leakage, higher survival rates, and better post-stress recovery capabilities. Physiologically, *VyMYB24* activates the antioxidant defense system to scavenge excess ROS, reduce MDA accumulation, and maintain membrane integrity and cellular homeostasis, thereby alleviating oxidative stress. These findings reveal that *VyMYB24* confers cold tolerance in a heterologous expression system through a multidimensional regulatory mechanism integrating morphological remodeling and antioxidant defense activation, highlighting the unique value of ‘Yanshan’ grape as a cold-resistant gene reservoir and providing a promising candidate gene for subsequent functional verification in grapevines and cold-tolerance molecular breeding.

## Data Availability

All data generated or analyzed during this study are included in this published article.

## Abbreviations

ROS: reactive oxygen species; SOD: superoxide dismutase; POD: peroxidase; CAT: catalase; MDA: malondialdehyde; DAB: 3,3′-diaminobenzidine; NBT: nitroblue tetrazolium; qRT-PCR: quantitative real-time polymerase chain reaction; WT: wild type; OE: overexpression; CBF: C-repeat binding factor; ICE1: inducer of CBF expression 1; GA: gibberellin; SD: standard deviation.
